# Computational application of internationally harmonized defined approaches to skin sensitization: DASS App

**DOI:** 10.1186/s12859-023-05617-1

**Published:** 2024-01-02

**Authors:** Kimberly T. To, Judy Strickland, Emily Reinke, Alexandre Borrel, Jim Truax, Heather Maldonado, Dave Allen, Nicole Kleinstreuer

**Affiliations:** 1 Predictive Toxicology and Information Sciences Group, Discovery and Safety Assessment Division, Inotiv, Inc., Morrisville, NC 27560 USA; 2 National Toxicology Program Interagency Center for the Evaluation of Alternative Toxicological Methods, Division of Translational Toxicology, National Institute of Environmental Health Sciences, National Institutes of Health, Research Triangle Park, NC 27709 USA

**Keywords:** Data integration, Computational prediction, Defined approaches, Skin sensitization, Non-animal methods

## Abstract

**Background:**

Chemically induced skin sensitization, or allergic contact dermatitis, is a common occupational and public health issue. Regulatory authorities require an assessment of potential to cause skin sensitization for many chemical products. Defined approaches for skin sensitization (DASS) identify potential chemical skin sensitizers by integrating data from multiple non-animal tests based on human cells, molecular targets, and computational model predictions using standardized data interpretation procedures. While several DASS are internationally accepted by regulatory agencies, the data interpretation procedures vary in logical complexity, and manual application can be time-consuming or prone to error.

**Results:**

We developed the DASS App, an open-source web application, to facilitate user application of three regulatory testing strategies for skin sensitization assessment: the Two-out-of-Three (2o3), the Integrated Testing Strategy (ITS), and the Key Event 3/1 Sequential Testing Strategy (KE 3/1 STS) without the need for software downloads or computational expertise. The application supports upload and analysis of user-provided data, includes steps to identify inconsistencies and formatting issues, and provides predictions in a downloadable format.

**Conclusion:**

This open-access web-based implementation of internationally harmonized regulatory guidelines for an important public health endpoint is designed to support broad user uptake and consistent, reproducible application. The DASS App is freely accessible via https://ntp.niehs.nih.gov/go/952311 and all scripts are available on GitHub (https://github.com/NIEHS/DASS).

**Supplementary Information:**

The online version contains supplementary material available at 10.1186/s12859-023-05617-1.

## Background

Skin sensitization testing is a critical step in regulatory chemical hazard assessment to determine the potential of a substance to cause an allergic reaction in humans following repeated dermal exposure. The historical standard animal-based (in vivo) tests used to determine skin sensitization potential are the mouse local lymph node assay (LLNA) and the guinea pig maximization test [1, 2]. Recently, non-animal cell-based (in vitro) and cell-free (in chemico) assays have been internationally adopted by regulatory authorities for predicting skin sensitization potential [3–5] and computational (in silico) models have been developed to predict skin sensitization based on chemical structure [6, 7]. However, the newly developed methods cannot be used as standalone replacements for in vivo animal studies and instead need to be combined with one another in testing strategies known as “defined approaches” (DAs).

A DA combines a specific set of information sources via a fixed data interpretation procedure to derive toxicity predictions. DASS have been developed to identify skin sensitizers by combining data from in vitro, in chemico, and in silico methods that map to the adverse outcome pathway (AOP) for skin sensitization [8]. An AOP is a conceptual representation of a biological network that links molecular and cellular key events (KE) to tissue and organismal-level adversity. An AOP can serve as a useful organizing framework for developing testing strategies based on the biology of the organism of interest and mechanistic knowledge [9].

DASS have demonstrated similar or better performance than the mouse LLNA when compared to reliable and reproducible human data [10]. In 2018, the U.S. Environmental Protection Agency (EPA) released a draft science policy that describes two DAs that they accept as replacements for animal tests to identify potential skin sensitizers: the 2 out of 3 (2o3) and the Key Event 3/1 Sequential Testing Strategy (KE 3/1 STS) [11–15]. Subsequently, in 2021 the Organisation for Economic Co-operation and Development (OECD) issued Guideline 497, which describes two validated DAs to identify potential skin sensitizers: 2o3 and the Integrated Testing Strategy (ITS) [14–16]. These DASS rely upon the *in chemico, in vitro*, and *in silico* information sources using human cells and defined molecular targets as well as chemical-structure based computational model predictions.

Given the regulatory acceptance of DASS, adoption of these methods is expected to increase. However, it can be challenging to implement DAs when evaluating multiple chemicals or to those unfamiliar with the composition and application of the DAs. In such cases, manual application of the data interpretation procedures can be time-consuming and prone to errors. Automated approaches can be used to apply the DAs more efficiently but require fluency in computational programming.

To address this challenge and facilitate broader adoption of these methods, we have developed the DASS App, an open-source, open-access web application that enables users to employ these validated non-animal approaches to evaluate chemical skin sensitization potential without the need for software downloads or computational expertise. The web application supports upload and analysis of user-provided data, includes steps to identify inconsistencies and formatting issues, and provides hazard and potency predictions in a downloadable format. The DASS App does not require the user to create an account. No data are retained by the application, which allows users to analyze data from substances in development without compromising their confidentiality.

### Implementation

The DASS App was developed with R v4.2.1 [17] using the *shiny* package [18]. The source code is available at https://github.com/NIEHS/DASS. The web application can be accessed at https://ntp.niehs.nih.gov/go/952311.

**Fig. 1:**
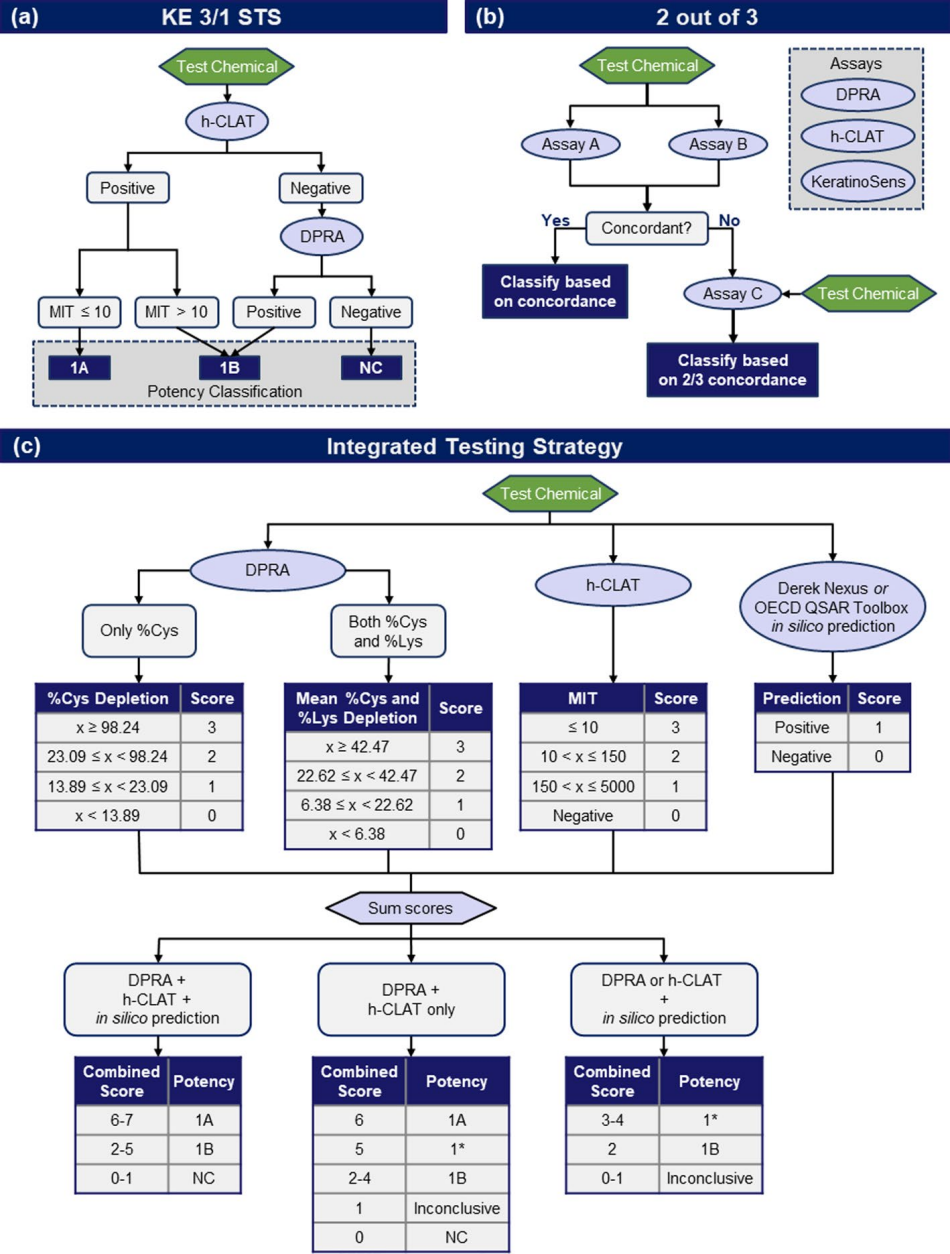
Defined approach data interpretation procedures. Potency categories are based on the GHS: 1A: strong sensitizer, 1B: weak sensitizer; NC: not classified (non-sensitizer). 1* indicates a conclusive sensitizer hazard prediction and an inconclusive potency prediction. **a** The KE 3/1 STS DA predicts skin sensitization hazard and potency by evaluating results from the human cell line activation test (h-CLAT) and direct peptide reactivity assay (DPRA). **b** The 2o3 DA predicts skin sensitization hazard by evaluating results from the DPRA, h-CLAT, and KeratinoSens. **c** The ITS DA predicts skin sensitization hazard and potency by scoring results from the DPRA, h-CLAT, and in silico predictions from Derek Nexus or the OECD QSAR Toolbox. The minimum induction threshold (MIT) is a quantitative endpoint from the h-CLAT. %-Cysteine (Cys) and %-Lysine (Lys) depletion are quantitative endpoints from the DPRA.

The DASS App allows users to apply three DAs: KE 3/1 STS, 2o3, and ITS [13–15]. The three DAs implement rule-based approaches for integrating multiple assay results to classify chemicals as sensitizers or non-sensitizers (hazard classification) (Fig. 1). The KE 3/1 STS, and ITS DAs will also predict chemical potency categories using the classifications established by the United Nations Globally Harmonized System of Classification and Labelling of Chemicals (GHS): strong sensitizer (1A), moderate/weak skin sensitizer (1B), and not classified (NC) [19].

Across the KE 3/1 STS, 2o3, and ITS DAs, three unique in vitro or in chemico information sources and two unique *in silico* information sources are used. The in vitro and in chemico assays each represent a KE in the AOP for skin sensitization [8]. The molecular initiating event of covalent interaction with skin proteins is addressed by the direct peptide reactive assay (DPRA) [3]. The second KE, keratinocyte activation, is addressed by the KeratinoSens™ assay (KS) [4], and the third KE of T-cell activation is addressed by the human cell line activation test (h-CLAT) [5]. Computational *in silico* predictions of skin sensitization potential generated by Derek Nexus software [6] or the OECD QSAR Toolbox [7] are used in the ITS.

The KE 3/1 STS DA is a sequential testing strategy that uses results from the DPRA and h-CLAT (Fig. 1a). First, the lowest concentration eliciting a positive outcome, or minimum induction threshold (MIT) from the h-CLAT, is evaluated to predict whether the chemical should be classified as a GHS 1A or 1B sensitizer; if the h-CLAT is positive, no further testing is required. However, if the h-CLAT is negative, DPRA results are evaluated to determine whether the chemical should be classified as a GHS 1B sensitizer or GHS NC (Fig. 1a). EPA currently accepts results from the KE 3/1 STS for hazard identification, and the DASS App also provides potency classification predictions [14, 15].

The 2o3 DA uses results from the DPRA, h-CLAT, and KS assays. Prediction of skin sensitization hazard is based on concordance of a sensitizer/non-sensitizer prediction from at least two of the three assays and is independent of any specified order in which to run each assay type. If two assays produce discordant results, then the third assay is necessary and the consensus result across the three assays is used to generate the hazard prediction (Fig. 1b). Otherwise, if there are only results from two assays and the results are discordant, the chemical cannot be classified, and the evaluation will return an “Inconclusive” result. The 2o3 DA does not predict GHS potency.

The ITS DA predicts skin sensitization hazard and GHS potency category by applying a scoring system to the mean % Cysteine (%Cys) and % Lysine (%Lys) depletion results from the DPRA, the MIT from the h-CLAT, and an *in silico* hazard prediction. The summed scores are used to predict chemical hazard and potency (Fig. 1c). The ITS DA includes multiple conditional scoring schemes to derive predictions in cases where the DPRA results only provide %Cys depletion or when data from only two of the information sources are available.

In the app, users upload a data file containing results from the information sources for their DA of interest. The DAs are applied to the user’s data using custom R functions (Additional file 1). The DA functions use the *fcase* function from the *data.table* package to apply the nested if-else logic within the data interpretation procedures.

## Results

The web application provides users with step-by-step modules to navigate through DA implementation to analysis of results. As users move from step to step, they are provided optional information boxes containing details and assistance for each specific step.

- The initial step requires users to select which DAs to implement.
- Users will then be prompted to upload their own data in tab-delimited, comma-delimited, or Excel format.
- In the next step, users specify the columns that correspond to the assay inputs required for their selected DAs. Manual column selection allows for flexibility of the order and names of the columns within user data and minimizes time spent on data preparation. Alternatively, users are provided a data template that can be used to prepare data *de novo*. When the data template is used, the app will automatically select data columns for the required assays, reducing the need for manual selection.
- After data selection, the DASS App evaluates the values in the user-selected columns against the data and formatting requirements and identifies any duplicate selections. If the DASS App identifies any errors in formatting or duplications, the column will be marked with a flag for the user to address.
- Data are then analyzed with the previously selected DAs with results immediately available. To assist user interpretation, the DASS App provides color-coded columns indicating selected data, translated and calculated input, and DASS predictions. The results can be downloaded as a tab-delimited or Excel file.
- Users may include reference data in their data file to compare against the DA predictions. Once the results are generated, users may specify the columns in their data that correspond to reference data and the DA predictions they want to evaluate. The DASS App will generate contingency tables and performance metrics for the user’s selected comparisons.

## Conclusions

The DASS App provides user-friendly, open access to validated non-animal testing strategies from international OECD guidelines and U.S. federal policies, advancing adoption across the scientific and regulatory communities and enabling consistent and reproducible computational data integration. Further development of the DASS App is ongoing to include evaluation of DA results against reference data from the Integrated Chemical Environment and facilitate physicochemical applicability domain analyses [20]. Efforts at the OECD to include assays analogous to the DPRA, KS, or h-CLAT, or other *in silico* methods, as alternative information sources within the DAs are ongoing. We plan to include additional assays and DAs as they are developed, validated, and accepted by regulatory authorities.

### Supplementary Information

**Additional file 1**. Defined approach R functions.

## Data Availability

Project name: The DASS App. Project home page: https://ntp.niehs.nih.gov/go/952311. Operating system(s): Platform independent. Programming language: R. Other requirements: Internet browser. License: MIT. Any restrictions to use by non-academics: None.

## Abbreviations

2o3: 2 out of 3
AOP: Adverse outcome pathway
DA: Defined approach
DASS: Defined approach for skin sensitization
EPA: Environmental Protection Agency
GHS: United Nations Globally Harmonized System of Classification and Labelling of Chemicals
ITS: Integrated Testing Strategy
KE 3/1 STS: Key Event 3/1 Sequential Testing Strategy
LLNA: Local lymph node assay
OECD: Organisation for Economic Co-operation and Development
